# The Remote Emerging Disease Intelligence—NETwork

**DOI:** 10.3389/fmicb.2022.961065

**Published:** 2022-09-02

**Authors:** Nicole L. Achee, The Remote Emerging Disease Intelligence—NETwork (REDI-NET) Consortium

**Affiliations:** ^1^Department of Biological Sciences, University of Notre Dame, Notre Dame, IN, United States; ^2^Eck Institute for Global Health, University of Notre Dame, Notre Dame, IN, United States

**Keywords:** *REDI-NET*, bio-/xeno-surveillance, emerging infectious disease, zoonoses, metagenomics, next-generation sequencing, mobile data entry, capacity building

## Abstract

Accurate prediction of zoonotic spillover events requires a detailed understanding of baseline pathogens circulating in differing global environments. By characterizing the diversity and determining the natural baseline of pathogens in a given biological system, any perturbations to this balance can be detected, leading to estimates of risk for emerging diseases. As epidemics and probability for pandemics increase, there is a fundamental need for building global collaborations to fill gaps in the surveillance effort, especially to build remote in-county capacity and standardize timely sample processing and data analysis. To this point, a new consortium, the Remote Emerging Disease Intelligence-NETwork (*REDI-NET*) has been established to enhance surveillance approaches and characterize natural pathogens in temperate, tropical forest, and tropical grassland biomes. The *REDI-NET* is envisioned to be a long-term, phased initiative. All phases will integrate accompanying training resources such as videos reflecting SOPs and Quick Reference Guides. Routine bio- and xenosurveillance will facilitate the characterization of ecological parameters, enhance the accuracy of vector species identification using artificial intelligence technology, and guide the establishment of epidemiological risk thresholds critical for mitigating disease outbreaks in a timely manner. A key deliverable of the *REDI-NET* is a custom-designed electronically merged (e-MERGE) data pipeline and alert dashboard that integrates remotely captured data with state-of-the-art metagenomic next-generation sequencing technology. This pipeline incorporates data generated from field and laboratory best practices, to furnish health decision-makers with a centralized, timely, and rigorous database to efficiently search interdisciplinary and heterogeneous data sources necessary to alert, prepare and mitigate health threats. The e-MERGE pipeline, once fully established, will be a flexible, scalable, and expandable tool for varied health applications. Program success will result in an operational framework that addresses resource gaps in pathogen surveillance and enhances health protection with broad global applicability. The objective of this manuscript is to introduce the REDI-NET framework to anticipated stakeholders engaged in metagenomics, epidemiological surveillance, and One Health with a focus on Phase 1.

## Introduction

An estimated 75% of emerging infectious diseases are zoonotic in nature, arising when pathogens in animals are passed to humans (Gebreyes et al., 2014), with vector-borne diseases accounting for more than 17% of all infectious diseases (World Health Organization, 2020). As the human population expands in number and into new geographical regions, the possibility that humans will come into close contact with animal species that are potential hosts of an infectious agent increases, including vector-borne diseases (VBD). Never before has the catastrophic impact of zoonotic spillover on humans needed less introduction. The World Health Organization (WHO) warned in its 2007 (World Health Organization, 2007) report that infectious diseases are emerging at a rate that has not been seen before. Since the 1970s, about 40 infectious diseases have been discovered, including SARS (Likhacheva, 2006), MERS (Lu and Liu, 2012), Ebola (Peterson et al., 2004), swine flu (Cohen and Enserink, 2009) and most recently SARS-CoV-2 (Sun et al., 2020). VBDs represent a major threat to public health worldwide. The re-emergence of dengue is recognized as a major concern by the WHO with more than 40% of the world’s population at risk and estimates of more than 300 million infections every year (World Health Organization, 2021). Chikungunya virus (CHIKV) recently received considerable attention due to outbreaks in the Indian Ocean, India, Europe, and Americas, with over 1 million cases recorded to date. After limited early outbreaks in the Pacific in 2007 and 2013, the Zika virus has spread to more than 30 countries in the Americas and the Caribbean, infecting over 100 million people (Moore et al., 2020). The recent rise of microcephaly cases and other neurological disorders reported in Brazil prompted the WHO to declare Zika as a Public Health Emergency of International Concern (WHO, 2017). Beyond these diseases that receive global attention, other VBDs of human health importance occur in the United States, such as West Nile Virus (World Health Organization, 2016) and Eastern Equine Encephalitis (Lindsey et al., 2015). In addition, across North America, the incidence of tick-borne diseases is increasing. The Center for Disease Control and Prevention (CDC) estimates that there are approximately 300,000 cases of Lyme disease (LD) annually, making LD the most common vector-borne disease in the U.S. (Molaei et al., 2015).

Universities can serve as important resources for technical training and education related to estimating disease threats and are leaders in the development of risk models and forecasting. Local health jurisdictions represent the first line of defense for preventing and controlling disease outbreaks and are tasked with carrying out limited surveillance activities in response to emerging pathogens; however, surveillance efforts are often narrow in scope (targeting a predetermined cohort of biological samples and testing for known pathogens) and reports can be seriously delayed if samples are sent to overloaded reach-back laboratories, taking weeks or months to be processed with data release occurring much later. Despite this heavy reliance on local authorities for a coordinated response to emerging pathogens, critical gaps exist in their ability to effectively do so. A recent evaluation found that local health personnel “expressed a lack of capacity to respond appropriately and effectively to emerging pathogens” (Center for Disease Control and Prevention, 2019). Respondents cited several areas of greatest weakness within their programs that included: (1) a lack of appropriate epidemiological surveillance tools (RESEARCH GAP), (2) a lack of adequately trained staff to carry out surveillance activities (TRAINING GAP), and (3) a lack of a uniform approach to data warehousing and analysis for risk assessments (COLLABORATION GAP).

The overarching aim of the *REDI-NET* is to develop a new U.S. and international laboratory consortium between academia and the Department of Defense (DoD) along with domestic and international partnering institutions to detect, predict, and mitigate emerging and re-emerging infectious disease threats and improve the accuracy and timeliness of the “data-to-decision” pipeline. Key expected deliverables include state-of-the-art standard operating procedures (SOPs), a functional e-MERGE data pipeline and *REDI-NET* database that includes custom-designed field data collection and web-based laboratory data collection applications, and a dashboard with built-in functionality to forecast emerging pathogens with user-friendly actionable outputs. Program success will result in an operational framework that addresses resource gaps in emerging disease surveillance and health protection with broad applicability.

Greater numbers of empowered remote laboratory and field personnel with a foundational background in monitoring and detecting pathogens will immediately benefit institutions and serve as local source guidance on appropriate surveillance methods, sample processing, and data usage to detect and mitigate potential zoonoses spill-over events. The computational infrastructure interface designed, built, and validated in this program will offer a broad range of tools for modeling data, simulations, data analysis, and the management of distributed data sources for health-decision making and future development of potential novel technologies such as vaccines and drugs.

## Materials and methods

The *REDI-NET* is envisioned to be a long-term, phased initiative (Table 1): Phase 1 leverages existing partnerships to provide a solid foundation of excellence to construct robust Standard Operating Procedures (SOPs) for field sampling and pathogen assessment in ticks, leeches, water and sediment and validate genomic outputs across laboratories; Phase 2 will expand upon environmental and invertebrate sentinel sample types, integrate stakeholder training, and optimize the platform through user feedback; while Phase 3+ will advance toward a One Health Approach with the inclusion of active animal sampling in new ecologies related to DoD Force Readiness; while the goal of Phase 4 is to roll the platform out to DoD Commands. All phases will integrate accompanying training resources such as videos reflecting SOPs and Quick Reference Guides.

**Table 1:** REDI-NET program scope.

Phase I (completed)	Aim 1: Establish robust SOPs for rigorous data capture and matched capabilities at *Gold* reach-back laboratories
	Aim 2: Active field surveillance across varied ecologies for broad-spectrum pathogen detection.
	Aim 3: Enable remote, verified *in-situ*, near real-time data acquisition for actionable reporting.
	Aim 4: Institute a data management pipeline for actionable reporting and threat forecasting.
Phase II (current)	Aim 1: Expand *REDI-NET* pathogen portfolio to include opportunistic vDNA outputs and an enhanced data warehouse.
	Aim 2: Develop an actionable workflow for early pathogen detection by remote forward-facing laboratories using the *REDI-NET* e-MERGE pipeline.
	Aim 3: Transfer knowledge of REDI-NET technologies and processes
Phase III: (planned)	Aim 1: Expand *REDI-NET* pathogen portfolio to include active vDNA sampling and field metadata collection for Force Health Protection / Force Readiness.
	Aim 2: Establish, maintain, and expand *REDI-NET* active surveillance across varied ecologies in existing and new Geographical Combatant Commands (GCCs) for broad-spectrum pathogen detection using established SOPs which include metagenomic approaches.
	Aim 3: Readiness for roll-out of the *REDI-NET* platform to Senior DoD Officials in six GCCs [NORTHCOM, SOUTHCOM, EUCOM, AFRICOM, CENTCOM, and INDOPACOM], and the AFHSC-GEIS partner network.

In each Phase, we will build new scientific collaborations and capacity by expanding consortium partnerships across institutions and bring the best HPDC practices to the utility of emerging infectious disease surveillance. A tiered laboratory schema will be applied throughout the initiative, whereby *Gold* Laboratories will serve as reach-back, verification centers and *Silver* Laboratories will serve as field-sample source locations. All laboratories will have matched capability for sample processing and testing.

**Figure 1:**
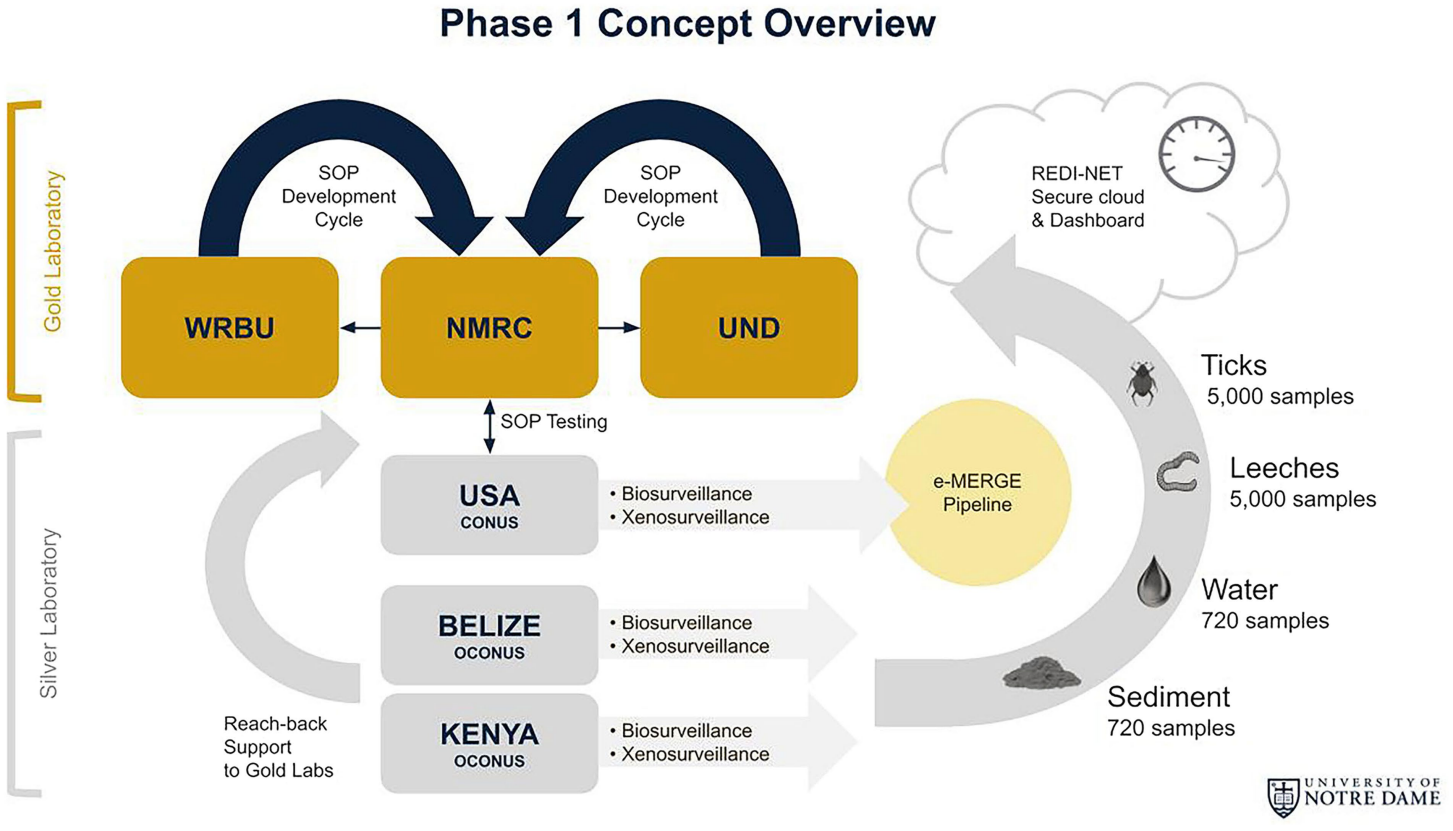
REDI-NET Phase I Concept Overview: (1) Establish robust SOPs for rigorous data capture and matched capabilities at Gold reach-back laboratories; (2) Active field surveillance across varied ecologies for broad pathogen catchment; (3) Enable remote, verified *in-situ*, near real-time data acquisition for actionable reporting; and (4) Institute a data management pipeline for actionable reporting and threat forecasting.

Phase 1 centers on enhanced surveillance approaches to characterize natural pathogens in select temperate [CONUS—Navy Entomology Centers of Excellence (NECE)], tropical forest (OCONUS—Belize), and tropical grassland (OCONUS—Kenya) sites. Sentinel sample types will include ticks, leeches, water, and sediment (Figure 1). These invertebrate and environmental sample types were selected based on their high probability of containing pathogens that permit total nucleic acid (TNA) extraction for metagenomic analyses. Tick surveillance conducted in Belize has shown a presence of tick-borne rickettsioses relevant to humans and animals (ASTHO, 2007). Several studies have documented the recovery of pathogens (Polsomboon et al., 2017; Gogarten et al., 2019) in water bodies, including watering holes and wastewater (Huver et al., 2015; Mosher et al., 2017; Alfano et al., 2021), and in larger invertebrate “blood-bags,” such as leeches (Herder et al., 2014; Huver et al., 2015; Mosher et al., 2017; Alfano et al., 2021). These results suggest that iDNA/eDNA-based surveillance approaches may complement efforts to proactively identify pathogens that could potentially spill over to humans or livestock. In addition, high-throughput automated AI technology for morphological tick species identification will be developed thus facilitating distance and real-time tick identification. The remote Tick e-ID platform (IDX) will be verified through the molecular identification of tick species.

The four specific aims in Phase 1 (Table 1):

**Aim 1**: Establish Robust Sops for Rigorous Data Capture and Matched Laboratory Capabilities.

To ensure consistent and reproducible pathogen detection results, a set of robust SOPs encompassing field collection, sample storage, laboratory processing and testing, and sample shipping have been developed, tested, optimized, and validated by all consortium laboratories through an iterative process. Corresponding digital data collection sheets (DCS) have also been developed from each SOP, which reflects the essential field and laboratory data required to capture throughout the *REDI-NET* e-MERGE pipeline. Training, and enabling of *Gold* and *Silver* laboratories through capacity strengthening to proficiently perform SOPs, has occurred either in-person or through remote sessions due to COVID-19 restrictions to ensure reproducibility of electronically sourced results from the remote locations using the *REDI-NET* e-MERGE pipeline. To maintain data rigor and identify any initial capability deficiencies, aliquots of reference materials containing the same mixture of bacteria (3 Gram-negatives and 5 Gram-positives including *Pseudomonas aeruginosa, Escherichia coli, Salmonella enterica, Lactobacillus fermentum, Enterococcus faecalis, Staphylococcus aureus, Listeria monocytogenes,* and *Bacillus subtilis*), viruses (Epstein–Barr virus and Human Immunodeficiency Virus 1), and fungi (*Saccharomyces cerevisiae* and *Cryptococcus neoformans*) organisms have been distributed to all current Consortium laboratories for output verification. Proficiency testing based on nanopore sequencing read number, length, quality, and coverage depth of each microbe has been conducted for current *Gold/Silver* individual operators and will be performed for any future laboratories joining the Consortium. This proficiency testing will be performed on an annual basis across the entire Consortium to ensure optimal and consistent data output from each partner laboratory. A second layer of verification has been implemented to have periodical submission of subsets of field-collected samples from *Silver* laboratories to the *Gold* reach-back laboratories to allow verification on the sequencing output and resultant microbe classification and abundance. In the event that human pathogens are detected, an Illumina sequencer equipped in all *Gold* laboratories will be used to confirm the initial findings from nanopore data.

**Aim 2**: Active Field Surveillance Across Varied Ecologies for Broad-Spectrum Pathogen Detection.

Tick collections are conducted monthly using dragging methodology in Belize, Florida, and Kenya from ecologically diverse areas of high animal footfall (i.e., woodland, forest-field margin, and around watering holes). Tick dragging is standardized across *REDI-NET Silver* field locations using a fixed sampling scheme, allowing aggregated analysis of presence/absence data along with densities and species composition. Ticks from domestic and/or wild animal collection based on the local approved protocols with the assistance of local veterinarians are also being processed as opportunistic samples. Standardized tick species identification capability in *Gold* laboratories will be developed from computer-vision, deep-learning algorithms using the IDX device (Brey et al., 2022) originally developed for mosquito species identification (Goodwin et al., 2021). Free-living leeches are being collected from water bodies using traps baited with beef liver (Ratnasingham and Hebert, 2013). Environmental samples (i.e., water and sediment) are being collected from the same Belize, Florida, and Kenya surveillance sites. Water and sediment samples are being gathered in triplicate using standard dip cups at each edge and 1 m into the water body. Invertebrates are kept alive, and both invertebrate and environmental samples are being transported to *Silver* laboratories at 4°C. All field samples will be stored at −80°C where possible or placed in the best RNA stabilizers (as determined by *REDI-NET* SOP development) or kept alive at 4°C (ticks, leeches; Herder et al., 2014; Mosher et al., 2017) until processing. Any samples shipped to the *Gold* labs for reach-back support and remote data validation will be shipped without breaking cold-chain and accompanied by the proper approved import/export permits according to the *REDI-NET* best practice shipping SOP. All specimens in each *Gold* and *Silver* laboratories are inventoried and entered into a custom-designed data management system.

**Aim 3**: Enable Remote, Verified *in-Situ*, Near Real-Time Data Acquisition for Actionable Reporting.

**Figure 2:**
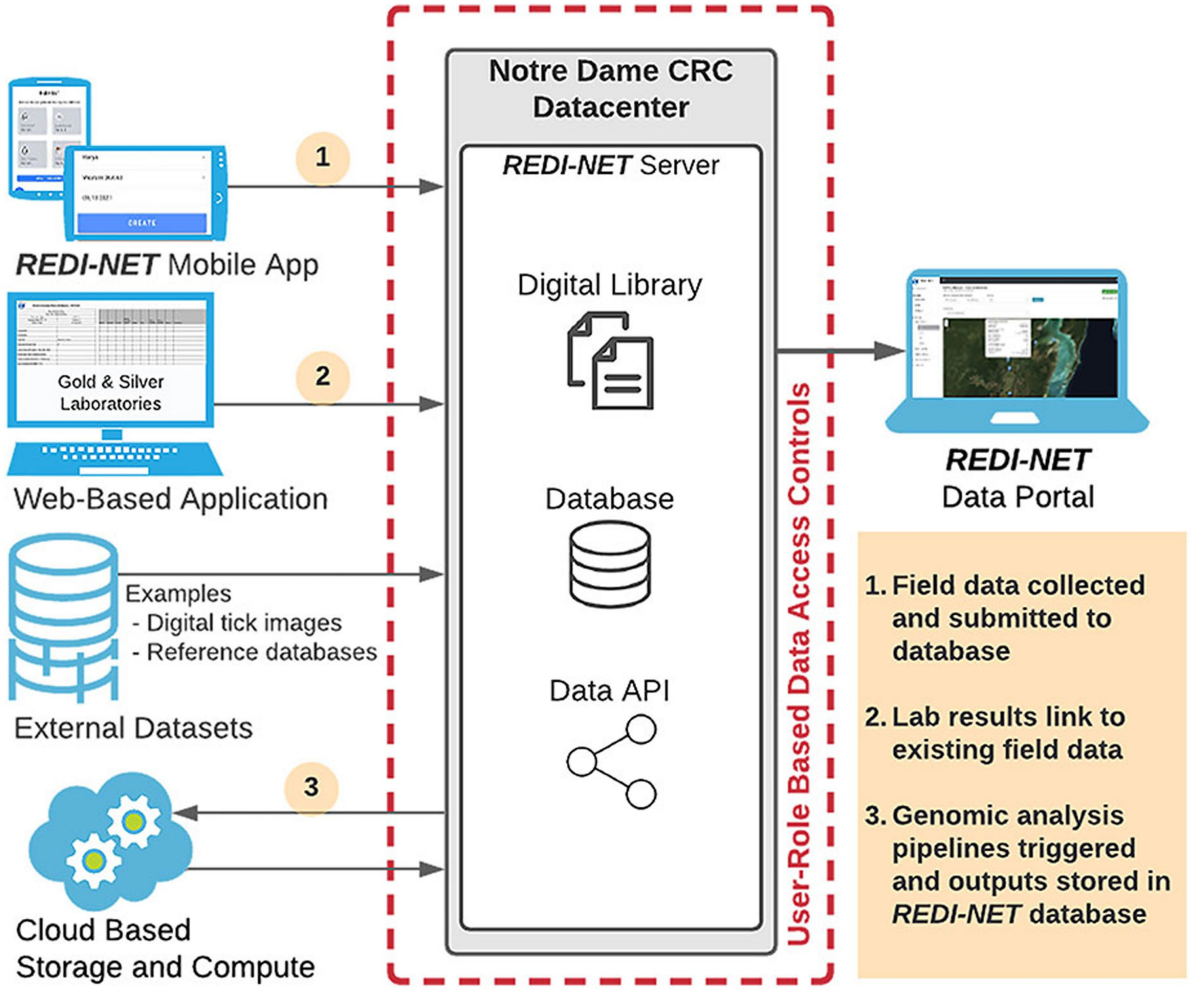
REDI-NET Phase I Data Management Structure.

A newly developed *REDI-NET* data warehouse with seamless integration of environmental and bio-/xeno-surveillance pathogen data will facilitate the development of the comprehensive e-MERGE pipeline, accessed through the newly established *REDI-NET* website (REDI-NET Consortium, 2022). The UND CRC team has developed and maintains a secure data repository to meet the *REDI-NET* project needs. The repository has been developed using modern web and database technologies to support data ingestion, validation, storage, and export for analysis (Figure 2).

An important aspect of the study will be the ability for *REDI-NET* consortium members to be able to monitor, access, and interact with the data and metadata being collected. The *REDI-NET* database allows dynamic data entry from each study site using a custom-designed mobile application for field data entry, hosted by CommCare, as well as a custom-designed web-based application for laboratory data entry providing the ability for near real-time data uploading and viewing. Critical functions of the *REDI-NET* data management system are provision of a digital library and data warehouse containing all available pathogen and vector-related data in a format that facilitates users to search and retrieve data and references pertaining to user-specified areas of interest. The data portal also hosts digital SOPs, Data Collection Sheets (DCSs), training videos, and reference materials. The digital library holds datasets of extracted parameter values for all primary vectors. Along with raw access to the data and metadata, the data portal will provide access to filterable visualizations of risk projections. Ultimately, the data portal provides decision-support tools and brings together the data, cutting-edge mathematical and statistical methods to improve emerging infectious disease modeling, validation, and prediction.

The *REDI-NET* mobile and web-based applications have been designed to assure data rigor based on SOPs. The DCSs were developed from the SOPs to establish quality control rules (i.e., drop-down lists, user entry fields) and inform the logic of the field data entry mobile application and *REDI-NET* database. In addition, the DCSs can be utilized if the field mobile application is unavailable all the while ensuring seamless data ingestion into the e-MERGE pipeline. The result will be a repository of both raw data and de-identified data, with accompanying metadata, immediately available for modeling, statistical analyses, and actionable reporting requirements. The data warehouse and file repository are backed up daily and weekly at the operating system level to ease recovery as needed. All data are stripped of personal identifiers (GPS information blurred) at the central data warehouse prior to any subsequent analysis and/or data sharing restrictions pursuant to DoD data sharing policies. Descriptive visualizations of pathogen occurrence or predicted trends in risk will be available *via* end-user interactive sessions with the database dashboard.

**Aim 4**: Institute a Data Management Pipeline for Actionable Reporting and Threat Forecasting.

A central function of the *REDI-NET* e-MERGE analysis pipeline will be the development of novel risk maps and models. Outputs from these models will be accessible to the end user *via* the *REDI-NET* dashboard. Expected modeling outputs include pathogen presence/absence estimates, seasonal projections of vector abundance and pathogen prevalence, and predictive disease and pathogen spillover risk maps. Surveillance data collected through *REDI-NET* will be combined with environmental and ecological data to generate predictive disease risk maps *via* a hierarchical Bayesian modeling framework. Such a framework enables the incorporation of multiple data types into the modeling process, including both historical and prospective data, both epidemiological and environmental data, and data collected at different spatial and temporal scales. At the same time, this modeling framework allows for the incorporation of associations between outcomes of interest and numerous spatial covariates, such as satellite-derived measures of temperature, wetness, land cover, and elevation. While some data—e.g., satellite-derived covariates, data collected prospectively by the REDI-NET—are available globally, others are unique to a given setting—e.g., historical surveillance data. The modeling framework allows for all such data types to jointly inform model-based predictions of pathogen risk.

Several types of model predictions will be generated. We will use the Bayesian modeling framework to map infection thresholds in the environment, and in arthropod and vertebrate animal populations. Among other available data types, the e-MERGE data analysis pipeline will allow us to integrate novel covariate datasets into these models. Surveillance data will be divided into training and testing sets, with the training data used to fit the models and the testing data used for model validation. Predicted infection thresholds from the model will then be resampled to generate predictive risk maps that can be used to estimate risk in unsampled locations. Where appropriate, we will also integrate point-based and polygon-based (areal) data into the predictive models using techniques to deal with the spatial misalignment of different datasets (Chilès and Delfiner, 2009; Rue et al., 2009). Last, our aim is to provide analytical tools that, once monitoring and surveillance become routine, can be used to investigate temporal trends to track changing pathogen patterns in the future. Spatial models will be extended to include a temporal component to capture seasonal patterns in pathogen prevalence and diversity. Spatiotemporal models can also be used to detect changes in pathogen distribution over time (seasonality) and to determine which ecological and environmental covariates are associated with any temporal trends in pathogen distribution, prevalence, or diversity (Christakos, 2000).

## Results

A total of 21 SOPs spanning field sampling, laboratory specimen processing, pathogen testing, specimen storage, shipment, and data management have been developed (Table 2). Current findings indicate that active field surveillance in Florida, Belize, and Kenya using custom-optimized *REDI-NET* SOPs has been capable of generating sequencing data leading to the detection of human pathogens in ticks, leeches, water, and soil. In addition, testing of reference material has generated consistent sequencing outputs indicating standardized capabilities throughout Consortium laboratories. Barcoding schemes have been developed to enable rigorous sample tracking and relating field metadata with laboratory pathogen testing outputs. The computer-vision algorithm for tick species identification has been developed from sample images taken with IDX submitted by all *Gold* laboratories for preliminary deployment in the IDX devices. The custom-designed *REDI-NET* database and both mobile field data entry and web-based lab data entry applications have been developed and verification of remotely captured data sync along with metadata relation functionality completed. The custom-developed *REDI-NET* technology (mobile/field, web-based/lab data entry applications) includes built-in functionality to ensure data integrity and data quality assurances. Additionally, quick reference guides and training videos have been developed for assuring standardization in methodology. A custom-designed *REDI-NET* sample storage web application has been integrated into the *REDI-NET* data portal to allow users to manage sample storage location for individual specimen records. A custom-designed *REDI-NET* dashboard and data portal have been designed as a resource repository to the database that hosts SOPs and DCSs, training videos, quick reference guides, equipment manufacturer data, and *REDI-NET* presentation templates with the ability to search/filter based on keywords. A NGS data upload mechanism has been established for the *REDI-NET* database and NGS software and data pipelines have been evaluated to identify those most appropriate for the *REDI-NET* pipeline. Risk models have been developed using publicly available databases (VectorMap and iNaturalist), published literature, and de-identified human case data from repositories to validate the mathematical model framework(s) for tick-borne disease risk estimation in Florida, Belize, and Kenya with model outputs available to visualize on the *REDI-NET* data portal. To ensure that the technical feasibility and viability of the *REDI-NET* framework is sustainable, and practical, as an early alarm of emerging infection threats, the Consortium will host end-user training sessions in each Phase to assess broad platform functionality and incorporate user feedback. Additionally, a digital *REDI-NET* resource package will be developed to facilitate ease of roll-out to global end users.

**Table 2:** Phase I REDI-NET standardized operating procedure (SOP) list.

No.	Title	Scope description
SOP T-1	Tick field sampling	To document the REDI-NET field processes for collecting samples
SOP L-1	Leech field sampling	
SOP W-1	Water field sampling	
SOP S-1	Sediment field sampling	
SOP T-2	Tick processing	To outline REDI-NET procedures to process samples for total nucleic acid extraction
SOP L-2	Leech processing	
SOP W-2	Water processing	
SOP S-2	Sediment processing	
SOP T-3	Tick storage	To outline steps for properly storing REDI-NET field-collected samples and nucleic acid samples purified from these samples
SOP L-3	Leech storage	
SOP W-3	Water storage	
SOP S-3	Sediment storage	
SOP T-4	Tick testing	To outline the REDI-NET procedures for properly using the Oxford Nanopore Sequencing platforms to sequence DNA and cDNA extracted from collected samples
SOP L-4	Leech testing	
SOP W-4	Water testing	
SOP S-4	Sediment testing	
SOP T-5	Tick shipping	To outline steps for proper packaging and shipping of preserved samples from a REDI-NET Silver lab to a REDI-NET Gold lab
SOP L-5	Leech shipping	
SOP W-5	Water shipping	
SOP S-5	Sediment shipping	
SOP DE	Data entry	To outline steps for properly recording and upload of data for the REDI-NET program

## Discussion

Epidemics of emerging and exotic nature are becoming more frequent and diverse worldwide and these outbreaks will inevitably continue into the foreseeable future (Gebreyes et al., 2014). Recently, the Washington Post Editorial Board presented a roadmap for living with COVID (Editorial Board, 2022) which highlighted gaps in infrastructure for monitoring and informing public policies, including the need for systematic surveillance. Often there is no overarching database architecture and/or platform that supports scalable approaches for building the necessary tools and services to facilitate the multi-user needs of emerging infectious disease surveillance data. This gap translates beyond COVID-19. Accurate prediction of zoonotic spillover events requires a detailed qualitative and quantitative understanding of the baseline environmental pathogens circulating in differing global land biomes, but detailed characterization regarding ecology, accurate species identification, and epidemiological thresholds, critical for actual success in mitigating disease outbreaks in a timely manner, has critical gaps. Any solution to alleviate these missing functional capabilities must also be cognizant of the underlying capacity, economic and sustainability issues at surveillance nodes.

The scientific premise of the *REDI-NET* was founded on the requirement of having tools and processes for near real-time, evidence-based, health decision-making with capacity strengthening for knowledgeable, competent health professionals and facilities for appropriate environmental pathogen surveillance, detection, and response. The *REDI-NET* has connected tangible, measurable partnerships amongst stakeholders to leverage expertise and resources for disease surveillance and management. The initial launch of the *REDI-NET* program has resulted in an operational framework that is meant to address some of the known resource gaps in infectious disease surveillance. The *REDI-NET* military/civilian collaborations have and will continue to broaden the reach of emerging disease surveillance activities for more accurate and timely predictive tools. We anticipate continued success in capacity-strengthening to occur as the *REDI-NET* initiative progresses. New consortium members will join the *REDI-NET*, remote data collection methodologies will be expanded using AI technologies, including drones for semi-autonomous and/or autonomous sample collection and we will consider adding machine learning algorithms to our spatial modeling platform, as these methods do not assume an underlying model and are robust to sampling biases (Elith et al., 2008; Alpaydin, 2020). Independent publications presenting from *REDI-NET* Phase 1 Aims are anticipated.

### REDI-NET Consortium (by organization then in alphabetical order)

#### University of Notre Dame

Nicole L. Achee, Department of Biological Sciences, Eck Institute for Global Health, University of Notre Dame, 239 Galvin Life Science Center, Notre Dame, IN, United States.

Maria Dahn, Department of Biological Sciences, University of Notre Dame, 239 Galvin Life Science Center, Notre Dame, IN, United States.

Benedicte Fustec, Department of Biological Sciences, University of Notre Dame, 265 Galvin Life Science Center, Notre Dame, IN, United States.

Miriam Grady, Department of Biological Sciences, University of Notre Dame, 301 Galvin Life Science Center, Notre Dame, IN, United States.

John P. Grieco, Department of Biological Sciences, Eck Institute for Global Health, University of Notre Dame, 243 Galvin Life Science Center, Notre Dame, IN, United States.

Donovan Leiva, Department of Biological Sciences, University of Notre Dame, 301 Galvin Life Science Center, Notre Dame, IN, United States.

Kara Linder, Department of Biological Sciences, University of Notre Dame, 301 Galvin Life Science Center, Notre Dame, IN, United States.

Sean Moore, Department of Biological Sciences, University of Notre Dame, 345 Galvin Life Science Center, Notre Dame, IN, United States.

Stacy Mowry, Department of Biological Sciences, University of Notre Dame, 347 Galvin Life Science Center, Notre Dame, IN, United States.

Alex Perkins, Department of Biological Sciences, Eck Institute for Global Health, University of Notre Dame, 347 Galvin Life Science Center, Notre Dame, IN, United States.

Caroline Pitts, Department of Biological Sciences, University of Notre Dame, 301 Galvin Life Science Center, Notre Dame, IN, United States.

Brooke Rodriguez, Department of Biological Sciences, University of Notre Dame, 301 Galvin Life Science Center, Notre Dame, IN, United States.

#### UND Center for Research Computing

Jaroslaw Nabrzyski, Center for Research Computing, University of Notre Dame, 829 Flanner Hall, Notre Dame, IN, United States.

Samuel Njoroge, Center for Research Computing, University of Notre Dame, 829 Flanner Hall, Notre Dame, IN, United States.

Matthew Noffsinger, Center for Research Computing, University of Notre Dame, 829 Flanner Hall, Notre Dame, IN, USA.

Kinsey Poland, Center for Research Computing, University of Notre Dame, 829 Flanner Hall, Notre Dame, IN, United States.

Caleb Reinking, Center for Research Computing, University of Notre Dame, 807 Flanner Hall, Notre Dame, IN, United States.

Bradley Sandberg, Center for Research Computing, University of Notre Dame, 829 Flanner Hall, Notre Dame, IN, United States.

#### Belize Vector and Ecology Center

Arlo Cansino, Belize Vector and Ecology Center, Slaughterhouse St, Orange Walk Town, Orange Walk, Belize, Central America.

Jailene Castillo, Belize Vector and Ecology Center, Slaughterhouse St, Orange Walk Town, Orange Walk, Belize, Central America.

Alvaro Cruz, Belize Vector and Ecology Center, Slaughterhouse St, Orange Walk Town, Orange Walk, Belize, Central America.

Marie C. Pott, Belize Vector and Ecology Center, Slaughterhouse St, Orange Walk Town, Orange Walk, Belize, Central America.

Uziel Romero, Belize Vector and Ecology Center, Slaughterhouse St, Orange Walk Town, Orange Walk, Belize, Central America.

#### Naval Medical Research Center

Tatyana Belinskaya, Naval Medical Research Center (NMRC), 503 Robert Grant Avenue, Silver Spring, MD 20910, United States; Henry M Jackson Foundation for the Advancement of Military Medicine, 6720A Rockledge Dr., Bethesda, MD, United States.

Erica Cimo, Naval Medical Research Center (NMRC), 503 Robert Grant Avenue, Silver Spring, MD 20910, United States; Henry M Jackson Foundation for the Advancement of Military Medicine, 6720A Rockledge Dr., Bethesda, MD, United States.

Le Jiang, Naval Medical Research Center (NMRC), 503 Robert Grant Avenue, Silver Spring, MD 20910, United States; Henry M Jackson Foundation for the Advancement of Military Medicine, 6720A Rockledge Dr., Bethesda, MD, United States.

Hsiao-Mei Liao, Naval Medical Research Center (NMRC), 503 Robert Grant Avenue, Silver Spring, MD 20910, United States; Henry M Jackson Foundation for the Advancement of Military Medicine, 6720A Rockledge Dr., Bethesda, MD, United States.

Zhiwen Zhang, Naval Medical Research Center (NMRC), 503 Robert Grant Avenue, Silver Spring, MD 20910, United States; Henry M Jackson Foundation for the Advancement of Military Medicine, 6720A Rockledge Dr., Bethesda, MD, United States.

#### Navy Entomology Center of Excellence

Jason Fajardo, Navy Entomology Center of Excellence, 937 Child St, Jacksonville, FL 32212, United States; Henry M Jackson Foundation for the Advancement of Military Medicine, 6720A Rockledge Dr., Bethesda, MD.

Edward Traczyk, Navy Entomology Center of Excellence, 937 Child St, Jacksonville, FL 32212, United States.

Melissa Vizza, Navy Entomology Center of Excellence, 937 Child St, Jacksonville, FL 32212, United States; Henry M Jackson Foundation for the Advancement of Military Medicine, 6720A Rockledge Dr., Bethesda, MD.

Christy Waits, Navy Entomology Center of Excellence, 937 Child St, Jacksonville, FL 32212, United States; Henry M Jackson Foundation for the Advancement of Military Medicine, 6720A Rockledge Dr., Bethesda, MD.

#### Walter Reed Biosystematics Unit

Brian Bourke, Walter Reed Biosystematics Unit (WRBU), Smithsonian Institution Museum Support Center, Suitland, MD, United States; One Health Branch, Center for Infectious Disease Research, Walter Reed Army Institute of Research (WRAIR), Silver Spring, MD, United States.

Laura Caicedo-Quiroga, Walter Reed Biosystematics Unit (WRBU), Smithsonian Institution Museum Support Center, Suitland, MD, United States; One Health Branch, Center for Infectious Disease Research, Walter Reed Army Institute of Research (WRAIR), Silver Spring, MD, United States.

Koray Ergunay, Walter Reed Biosystematics Unit (WRBU), Smithsonian Institution Museum Support Center, Suitland, MD, United States; One Health Branch, Center for Infectious Disease Research, Walter Reed Army Institute of Research (WRAIR), Silver Spring, MD, United States; Virology Unit, Department of Medical Microbiology, Faculty of Medicine, Hacettepe University Ankara, Turkey.

Yvonne-Marie Linton, Walter Reed Biosystematics Unit (WRBU), Smithsonian Institution Museum Support Center, Suitland, MD, United States; One Health Branch, Center for Infectious Disease Research, Walter Reed Army Institute of Research (WRAIR), Silver Spring, MD, United States; Department of Entomology, Smithsonian Institution National Museum of Natural History, Washington, DC, United States.

Suppaluck Nelson, Walter Reed Biosystematics Unit (WRBU), Smithsonian Institution Museum Support Center, Suitland, MD, United States; One Health Branch, Center for Infectious Disease Research, Walter Reed Army Institute of Research (WRAIR), Silver Spring, MD, United States.

David B. Pecor, Walter Reed Biosystematics Unit (WRBU), Smithsonian Institution Museum Support Center, Suitland, MD, United States; One Health Branch, Center for Infectious Disease Research, Walter Reed Army Institute of Research (WRAIR), Silver Spring, MD, United States.

Alexander Potter, Walter Reed Biosystematics Unit (WRBU), Smithsonian Institution Museum Support Center, Suitland, MD, United States; One Health Branch, Center for Infectious Disease Research, Walter Reed Army Institute of Research (WRAIR), Silver Spring, MD, United States.

Dawn Zimmerman, Walter Reed Biosystematics Unit (WRBU), Smithsonian Institution Museum Support Center, Suitland, MD, United States; Department of Epidemiology of Microbial Disease, Yale School of Public Health, New Haven, CT, United States.

#### Smithsonian Institution

James Hassell, Global Health Program, Smithsonian’s National Zoo and Conservation Biology Institute, Washington, DC, United States; Department of Epidemiology of Microbial Disease, Yale School of Public Health, New Haven, CT, United States.

#### Mpala Research Centre

Maureen Kamau, Mpala Research Centre, Laikipia, Kenya; Global Health Program, Smithsonian’s National Zoo and Conservation Biology Institute, Washington, DC, United States.

Rashid Lebunge, Mpala Research Centre, Laikipia, Kenya.

Janerose Mutura, Mpala Research Centre, Laikipia, Kenya.

#### George Mason University

Michael von Fricken, George Mason University, 4,400 University Drive, Fairfax, VA, United States.

Abigail A. Lilak, George Mason University, 4,400 University Drive, Fairfax, VA, United States.

Graham A. Matulis, George Mason University, 4,400 University Drive, Fairfax, VA, United States.

#### Vectech

Jewell Brey, Vectech, 3,600 Clipper Mill Rd., STE 205, Baltimore MD, United States.

Tristan Ford, Vectech, 3,600 Clipper Mill Rd., STE 205, Baltimore MD, United States.

Adam Goodwin, Vectech, 3,600 Clipper Mill Rd., STE 205, Baltimore MD, United States.

Ghnana Madineni, Vectech, 3,600 Clipper Mill Rd., STE 205, Baltimore MD, United States.

Bala Murali Manoghar Sai Sudhakar, Vectech, 3,600 Clipper Mill Rd., STE 205, Baltimore MD, United States.

Sanket Padmanabhan, Vectech, 3,600 Clipper Mill Rd., STE 205, Baltimore MD, United States.

#### Former consortium members (no longer actively affiliated)

Robert Ang’ila, Mpala Research Centre, Laikipia, Kenya.

Margaret Elliott, Department of Biological Sciences, University of Notre Dame, 301 Galvin Life Science Center, Notre Dame, IN, United States.

Joanna Gomez, Belize Vector and Ecology Center, Slaughterhouse St, Orange Walk Town, Orange Walk, Belize.

Lauren Maestas, Navy Entomology Center of Excellence, 937 Child St, Jacksonville, FL, United States.

Marla S. Magaña, Belize Vector and Ecology Center, Slaughterhouse St, Orange Walk Town, Orange Walk, Belize.

Mohamed Sallam, Navy Entomology Center of Excellence, 937 Child St, Jacksonville, FL, United States.

Alexia Thompson, Belize Vector and Ecology Center, Slaughterhouse St, Orange Walk Town, Orange Walk, Belize.

## Data availability statement

The datasets presented in this article are not readily available because REDI-NET Phase 1 data generation and analysis is ongoing with associated datasets to become available at the time specific outcomes are published. Requests to access the datasets should be directed to NA, redinet@nd.edu.

## Author contributions

The REDI-NET was conceived by JPG, LJ, Y-ML, and NA. LJ and H-ML led the development of laboratory sample processing and testing SOPs. NA, JPG, LJ, Y-ML, BF, MD, and CP contributed to the development of field sampling, storage, and shipment SOPs. Gold laboratory data are being generated by BF, DL, CP, H-ML, TB, ZZ, EC, BB, LC-Q, KE, SN, DP, and APo. Silver field and laboratory data are being generated in Belize by ACa, JC, ACr, MP, and UR, in Florida by ET, JF, MV, and CW, and in Kenya by DZ, JH, MK, RL, JM, MF, AL, and GM. Management of the central data repository is led by JN, SN, MN, KP, CR, and BS. Mathematical modeling is being conducted by APe, SMoo, and SMow. Literature searches were conducted by ME, BR, and MG. Human and animal use applications are managed by NA. Tick e-ID platform development led by TF, AG, JB, GM, BS, and SP. The first draft of the manuscript was written by JG, LJ, Y-ML, MF, MD, BS, APe, SMoo, SMow, and NA. All authors contributed to the article and approved the submitted version.

## Funding

This work was supported by the United States Army Medical Research and Development Command under contract no. W81XWH-21-C-0001.

## Conflict of interest

The author declares that the research was conducted in the absence of any commercial or financial relationships that could be construed as a potential conflict of interest.

## Publisher’s note

All claims expressed in this article are solely those of the authors and do not necessarily represent those of their affiliated organizations, or those of the publisher, the editors and the reviewers. Any product that may be evaluated in this article, or claim that may be made by its manufacturer, is not guaranteed or endorsed by the publisher.

## Author disclaimer

The views, opinions, and/or findings contained in this report are those of the author(s) and should not be construed as an official Department of the Army position, policy, or decision unless so designated by other documentation. The views expressed in this article are those of the author and do not necessarily reflect the official policy or position of the Department of the Navy, Department of Defense, nor the U.S. Government.
